# Evaluating Children with Otitis Media for Bacteremia or Urinary Tract Infection

**DOI:** 10.1155/2010/790167

**Published:** 2010-08-16

**Authors:** Daniel Yawman, Patrick Mahar, Aaron Blumkin, Gregory Conners

**Affiliations:** ^1^Department of Pediatrics and Emergency Medicine, University of Rochester Medical Center and Rochester General Hospital, Box 238 1425 Portland Avenue, Rochester, NY 14621, USA; ^2^Emergency Medicine, Denver Children's Hospital, Denver, CO 80045, USA; ^3^Department of Pediatrics, University of Rochester Medical Center and Rochester General Hospital, Rochester, NY 14642, USA; ^4^Department of Pediatrics, Children's Mercy Hospital & Clinics, Kansas City, MO 64108, USA

## Abstract

*Background.* It is unclear if clinicians evaluate for concurrent bacteremia or UTI in young patients diagnosed with acute otitis media (AOM). *Objectives.* To describe how often, and under which circumstances, emergency providers investigate for bacteremia or UTI in 2–36 month olds with AOM. *Methods.* Cases of AOM were analyzed from the 2001–2004 National Hospital Ambulatory Medical Care Survey (NHAMCS)-Emergency Department dataset. *Results.* AOM was diagnosed in 17% of the 10,847 recorded visits of 2–36 month olds. Of these visits, laboratory testing included: CBC: 7%, Blood culture: 4%, urinalysis or urine culture: 5%, and any of these tests: 9%. Rates of testing for 2–6 month olds with temperature ≥ 38.0 (CBC: 13%, blood culture: 9%, urinalysis or urine culture: 7%, any of the tests: 14%) were not significantly different from testing of patients aged 6–12 months, or 12–36 months (all *P* > .1). Patients with temperature of ≥39.0 were more likely to have all tests, with the exception of urine investigation, than patients with temperature between 38.0 and 38.9. *Conclusions.* 17% of 2–36 month old patients seen in the emergency department are diagnosed with AOM. Investigating for bacteremia or UTI in these patients is not routine, even in febrile infants.

## 1. Introduction

Acute otitis media (AOM) is the most common infection for which antibiotics are prescribed for children in the United States [1]. There were 2.6 million emergency department visits for AOM in 1996, and 2.1 million visits in 2005 [2]. AOM is often associated with fussiness, otalgia, and fever, but these signs and symptoms are nonspecific and are associated with other childhood infections [1]. Although young children with fever commonly have AOM or a viral illness, some febrile children have occult bacterial infections, such as bacteremia or urinary tract infection (UTI).

*Streptococcus pneumoniae* is a known cause of AOM and bacteremia in children. Data published prior to the introduction of the pneumococcal conjugate vaccine (PCV) in February 2000 showed that rates of bacteremia in febrile 3–36-month-olds diagnosed with AOM were similar to those without a source of fever [3]. Another study showed that febrile 3–36-month-olds with a bacterial source of infection (including AOM) had similar rates of bacteremia as those without a focal bacterial source of their fever [4].

The PCV has been associated with a moderate decrease in the overall incidence of AOM [5–8]. Many studies have also documented a decrease in *streptococcus pneumoniae* bacteremia since the introduction of the PCV in February, 2000 [9–14]. One study involved the evaluation of 2–36-month-olds with fever ≥ 39.0 who presented to either emergency departments or urgent care centers, excluding patients who had a focal bacterial source of infection, other than AOM. The rate of bacteremia in this population was 0.9% [15]. Another study showed that 0.9% of all febrile patients' ages 57–180 days presenting to the emergency department were bacteremic. This same study reported that the incidence of urinary tract infection (UTI) was 9.7% [16]. In another study which included children up to two years of age, 5.9% were found to have UTI in those without a source of fever, and 2.7% of those with AOM or other source of fever had a UTI [17]. These studies suggest that in those with AOM the incidence of UTI is low, and the incidence of bacteremia is rare. 

The main objective of this study is to characterize the extent that emergency practitioners investigate for bacteremia or UTI in 2–36-month-old patients diagnosed with AOM in the four years following the introduction of the PCV. A second goal is to identify if temperature or age are associated with higher rates of testing. Whether a complete blood count (CBC) is performed as part of the laboratory investigation will also be described as this test has been used to predict bacteremia [18, 19].

## 2. Methods

### 2.1. Study Design

We analyzed data from The National Hospital Ambulatory Medical Care Survey (NHAMCS)-Emergency Department from 2001–2004 [20]. The study was ruled exempt from informed consent by the local institutional review committee.

### 2.2. Study Setting and Population

The National Hospital Ambulatory Medical Care Survey (NHAMCS) is designed to collect data on the utilization and provision of ambulatory care services in hospital emergency and outpatient departments in the United States. The NHAMCS dataset is a nationally representative multistage stratified probability sample of visits to the emergency departments and outpatient departments of noninstitutional general and short-stay hospitals within the United States, excluding federal, military, and Veterans Administration hospitals. The study is administered by the Division of Health Care Statistics, Centers for Disease Control and Prevention. Only data regarding emergency visits were included in this study.

### 2.3. Study Protocol

Interviewers visit the hospitals prior to their participation in the survey to explain survey procedures, verify eligibility, develop a sampling plan, and train hospital staff in data collection procedures. The survey instrument is the patient record form. Hospital staff is instructed to complete patient record forms for a random sample of patient visits during a randomly assigned 4-week reporting period. The U.S. Bureau of the Census acts as the field data collection agent for the NHAMCS.

### 2.4. Measurements

A case of AOM was identified if the International Classification of Diseases, Ninth Revision (ICD-9) code of 382.9 was noted in the primary or secondary diagnosis. If AOM was identified as a secondary diagnosis, the ICD-9 code for the primary diagnosis was identified. Demographic data and primary reason for visit were recorded for each case of AOM. Boxes on the data form were checked if a complete blood count (CBC), blood culture, urine culture, and urinalysis were performed at the visit. Whether an electrocardiogram (ECG), electroencephalogram (EEG), magnetic resonance image (MRI), or Computerized Axial Tomography scan (CT) was obtained was recorded in a similar fashion. Final disposition of the patient was also identified for each case. For purposes of analysis, the term “urine investigation” was used to define a patient that had either a urinalysis or a urine culture performed. The term “any testing” was used to describe a patient who had at least one of the following; CBC, blood culture, or urine investigation.

### 2.5. Data Analysis

We utilized four years of data (2001–2004). These four years were chosen for study as each of the essential data fields (acute otitis media diagnosis, temperature, CBC, blood culture, urinalysis, urine culture) were obtained similarly with no alteration in the yearly survey instrument. Alterations in the methodology of data collected after 2004 from the previous year's methodology prevented inclusion of years beyond 2004. 

 Patients were included if their primary or secondary diagnosis was AOM. Patients were excluded from the study if temperature was not recorded or if investigation for an alternative diagnosis was undertaken (ECG, EEG, MRI/CT). Patients were also excluded if the disposition was admission to a hospital or transfer to another facility. Each of these circumstances was thought to represent a population of children that had a more complex clinical presentation. Therefore, after exclusions, the expectation was that the sample would be comprised of children with uncomplicated AOM that was managed as an outpatient. 

Bivariate associations using chi-square analyses were performed to assess laboratory testing performed by temperature stratified by age (2–6 months versus 6–12 months versus 12–36 months). The proportion of subjects who received each laboratory test was compared between each of the four years of data to identify trends in laboratory investigation over the duration of the study. Multivariable logistic regression analyses were performed on febrile patients to predict laboratory testing based on temperature, age, gender, insurance type (private, Medicaid/SCHIP, Self-pay, or other), and race/ethnicity (white, black, Hispanic, or other). Stata version 10.1 (Stata Corp, College Station, Texas) was used to adjust for the complex sampling design.

## 3. Results

Data were collected from 10,847 emergency department visits of 2–36-month-old patients from 2001–2004. 17% of these patients received a primary (13%) or secondary (4%) diagnosis of AOM. Further analyses were performed on these 17% diagnosed with AOM. Of these patients, 3% were excluded because temperature was not recorded. Another 0.9% was excluded because they were either admitted to the hospital or transferred to another hospital. Less than 0.01% was excluded for having an ECG, EEG, or MRI/CT. After exclusions, 96% of all 2–36-month-old patients diagnosed with AOM remained in the study sample for further analyses (*n* = 1881). Characteristics of the study sample are shown in Table 1. Of note, 57% of the children who had a diagnosis of AOM had a triage temperature less than 38.0°C. Of those included in the study with a secondary diagnosis of AOM, the primary diagnosis is listed in Table 2. 

Overall, 7% had a CBC, 3% had a blood culture, 4% had a urinary investigation, and 9% had any of these laboratory tests. Overall trends of obtaining laboratory testing (CBC, blood culture, urine investigation, or any testing) did not vary significantly from year to year during the four years of data collection (all *P* > .1 for each test). Likewise, laboratory testing rates for each test were not statistically different between those with a primary diagnosis of AOM compared to those with a secondary diagnosis of AOM after adjusting for fever and multiple comparisons (all *P* ≥ .1). Rates of each laboratory test stratified by age group and temperature are shown in Table 3. Overall rates of obtaining individual tests, or any testing, in patients with temperature <38.0 were extremely low. Rates of testing were not significantly different between the three age groups in patients with temperature ≥ 38.0. Rates of testing were also evaluated for infants with temperature ≥ 39.0. Due to a low sample size of 2–6-month-olds with temperature ≥39.0, 2–6-month and 6–12-month-olds were combined for analyses. In 2–12-month-olds with temperature ≥ 39.0 rates of testing included: CBC 19%, blood culture 10%, urine investigation 12%, and any testing 24%.

Multivariable analyses were performed on only those patients with a diagnosis of AOM and a temperature of ≥ 38.0 (Table 4). Patients with temperature of ≥ 39.0 were more likely to have all tests, with the exception of urine investigation, than patients with temperature between 38.0 and 38.9. In addition, there were no statistically different rates of testing between young infants (2–6 months) and older infants (6–12 months), or toddlers (12–36 months). Gender, insurance type, and race/ethnicity were included in the model, but were not found to be significantly associated with laboratory testing. 

## 4. Discussion

The diagnosis of AOM is common: 17% of all 2–36-month-olds presenting to emergency departments in the United States are diagnosed with AOM. Nearly all of these patients are discharged home. A previous publication from this dataset noted that antibiotics are prescribed to a majority of these children [2]. This study aimed to describe the extent that clinicians evaluate for bacteremia or UTI in patients diagnosed with AOM in the emergency department. 

This study helps to clarify that evaluating for bacteremia or UTI is not routinely pursued in 2–36-month-olds diagnosed with AOM in the emergency department. It is of note, however, that approximately one quarter of all 2–12-month-olds with fever ≥39.0 had a CBC, blood culture or urine investigation performed. Investigating for bacteremia was nearly as common as investigating for a urinary source of infection. Previous data suggest that rates of urinary infections are much higher than rates of bacteremia, but our results do not suggest higher vigilance in investigating a urinary source of infection over bacteremia. It is possible that there is a perception that although UTI is more common, bacteremia is more serious, and therefore should be investigated more thoroughly. There also exists the possibility that a urinary catheter is viewed as a more invasive procedure than a venipuncture, and therefore clinicians are less likely to catheterize a young patient. 

It is unclear if other factors lead certain clinicians to pursue laboratory testing in young patients with AOM. It is conceivable that some clinicians may have uncertainty distinguishing between otitis media with effusion (OME) and AOM. Previous literature has shown that clinicians often misdiagnose OME as AOM [21, 22]. Perhaps clinicians who diagnose AOM, but do so with uncertainty, are more likely to aggressively evaluate the patient as they view this patient as one without a source of infection. Further research could investigate if clinicians base their decision to perform laboratory testing on their confidence level of their diagnosis of AOM. Another possibility is that despite a confident diagnosis of AOM, a clinician may still feel the risk of bacteremia or UTI is sufficient to warrant testing. This management would be supported by previous literature suggesting that a focal infection decreases, but does not eliminate, the possibility of bacteremia or UTI [16]. 

The primary strength of this study is that it is based on a national sample of infants and children presenting to emergency departments. This reflects care of children both within and outside of children's hospitals. This study is relevant to all clinicians who treat children as AOM is the most common infection for which antibiotics are prescribed in childhood. 

## 5. Limitations

There are limitations to this study. The most important of these is that there is no knowledge of the results of the laboratory testing that was performed. Therefore, this paper does not address whether clinicians *should* evaluate for bacteremia or UTI in patients diagnosed with AOM. These data only provide evidence that testing is not routine, even in febrile younger patients. Also, it would have been helpful to describe the percentage of young infants (2–6 months of age) with temperature ≥39.0 who receive a laboratory evaluation. Our data demonstrate that AOM is not as common in 2–6-month-olds as it is in older infants. Therefore, there was an inadequate sample of 2–6-month olds with temperature ≥39.0 to make accurate comparisons to those with a temperature of 38.0-39.0, or to those between 6–12 months of age. The multivariable analyses do suggest that 2–6-month-olds do not receive more testing than 6–12-month-olds, but further study would be needed to accurately describe testing rates of 2–6-month-olds with fever ≥39.0.

Also, when a urinalysis was ordered by a clinician, the intention of the urinalysis could not be determined from these data. For example, a urinalysis may be performed to assess for dehydration or to investigate for an infection. There is no mechanism to know from these data why the test was ordered. We assumed that anytime a urinalysis or a urine culture was performed, the clinician was investigating for infection. Therefore, we may be overestimating the number of clinicians who were investigating for a UTI. 

Lastly, whether or not a provider was aware of the PCV vaccine status of the participants in the study is not noted in these data. This would have helped clarify if clinical decisions were based on the vaccination status of the participant. Much of the medical literature documenting the clinical decrease in rates of *streptococcus pnuemoniae* bacteremia due to the PCV was not available in 2001–2004. Therefore, even if providers were aware that a participant received the PCV, it is possible that providers may have based clinical management upon their knowledge of the higher rates of *streptococcus pnuemoniae* bacteremia that existed prior to the PCV licensure in 2000. As such, providers in this study may have been more likely to evaluate for bacteremia than those in the years following the study.

## 6. Conclusion

From 2001–2004, 17% of 2–36-month-olds who presented to emergency departments in the United States were diagnosed with AOM. Evaluating for bacteremia or UTI was not routinely performed. Less than one in four infants with AOM and fever ≥ 39.0 was investigated for these occult infections.

## Figures and Tables

**Table 1:** Description of patients diagnosed with acute otitis media.

Gender	
Male	55%
Female	45%
Age	
2–6 months	10%
6–12 months	28%
12–35 months	62%
Race/Ethnicity	
White (non-Hispanic)	49%
Hispanic	24%
Black (non-Hispanic)	23%
Other	4%
Temperature at Triage	
Less than 38.0	57%
38.0–38.9	21%
Above 39.0	22%
Insurance	
Private Insurance	32%
Medicaid/SCHIP	53%
Self-pay	7%
Other	7%
Chief Complaint	
Fever	38%
Respiratory*	18%
Ear complaint^#^	16%
Gastrointestinal^+^	7%
Rash	4%
Cranky/Fussy	2%
Other	15%

*Includes upper respiratory infection, cough, or nasal congestion.

^#^Includes ear pain, pulling ear, ear discharge, ear infection.

^+^Includes vomiting, nausea, and diarrhea.

**Table 2:** Primary diagnosis of those with a secondary diagnosis of acute otitis media.

Diagnosis	%
(1) Viral*	31
(2) General symptoms or fever	25
(3) Bronchitis/bronchiolitis	9
(4) Asthma	6
(5) Gastroenteritis/digestive symptoms	6
(6) Pharyngitis/tonsillitis/laryngitis	5
(7) Rash/Skin condition	4
(8) Pneumonia	3
(9) Conjunctivitis	2
(10) Ear pain/otitis externa	2
(11) Febrile seizure	2
(12) Sinusitis	1
(13) Other	5

*Viral includes diagnoses of influenza, common cold, viral illness, upper respiratory infection, respiratory illness, and croup.

**Table 3:** Laboratory evaluation of 2–36-month-old patients with acute otitis media stratified by temperature and age in months.

	<38.0				≥38.0			
Test	2–6	6–12	12–36	*P-Value	2–6	6–12	12–36	*P-Value
CBC	5%	3%	3%	.41	13%	15%	13%	.75
Blood Culture	3%	1%	1%	.02	9%	8%	6%	.64
Urine Investigation	3%	2%	1%	.09	7%	11%	6%	.12
Any of the Above	5%	4%	3%	.51	14%	20%	20%	.34

*All *P*-Values denote differences between three age categories.

**Table 4:** Adjusted odds ratios (OR) from multivariable logistic regression for obtaining laboratory tests in patients with acute otitis media and temperature ≥38.0.

	CBC		Blood culture		Urine investigation		Any testing	
	OR (95% CI)	*P*-value	OR (95% CI)	*P*-value	OR (95% CI)	*P*-value	OR (95% CI)	*P*-value
Age								
2–6 months	1.0 [reference]		1.0 [reference]		1.0 [reference]		1.0 [reference]	
6–12 months	1.0 (0.4–2.4)	.98	0.8 (0.2–2.6)	.72	1.5 (0.5–4.9)	.45	1.3 (0.6–3.0)	.51
12–36 months	0.8 (0.3–2.0)	.66	0.6 (0.2–2.0)	.42	0.8 (0.2–2.6)	.72	1.0 (0.4–2.3)	.96
Temperature °C								
38.0–38.9	1.0 [reference]		1.0 [reference]		1.0 [reference]		1.0 [reference]	
≥39.0	** 2.0 (1.2–3.4)**	**.01**	** 2.4 (1.2–4.7)**	**.01**	1.5 (0.8–3.0)	.22	** 1.7 (1.0–2.9)**	**.05**
